# Supplementary material for: The Evaluation of Drugs as Potential Modulators of the Trafficking and Maturation of ACE2, the SARS-CoV-2 Receptor
Source: Biomolecules. 2024 Jun 27;14(7):764. doi: 10.3390/biom14070764 (PMC11274373; doi:10.3390/biom14070764)
Supplement: Supplementary file 1 [file biomolecules-14-00764-s001.zip › biomolecules-3041240-original-images.pdf]

Results figures
+ blots(not for publication)

Paper title: The evaluation of drugs as potential modulators of the trafficking and maturation of ACE2, the SARS-CoV-2 receptor

Figure1:

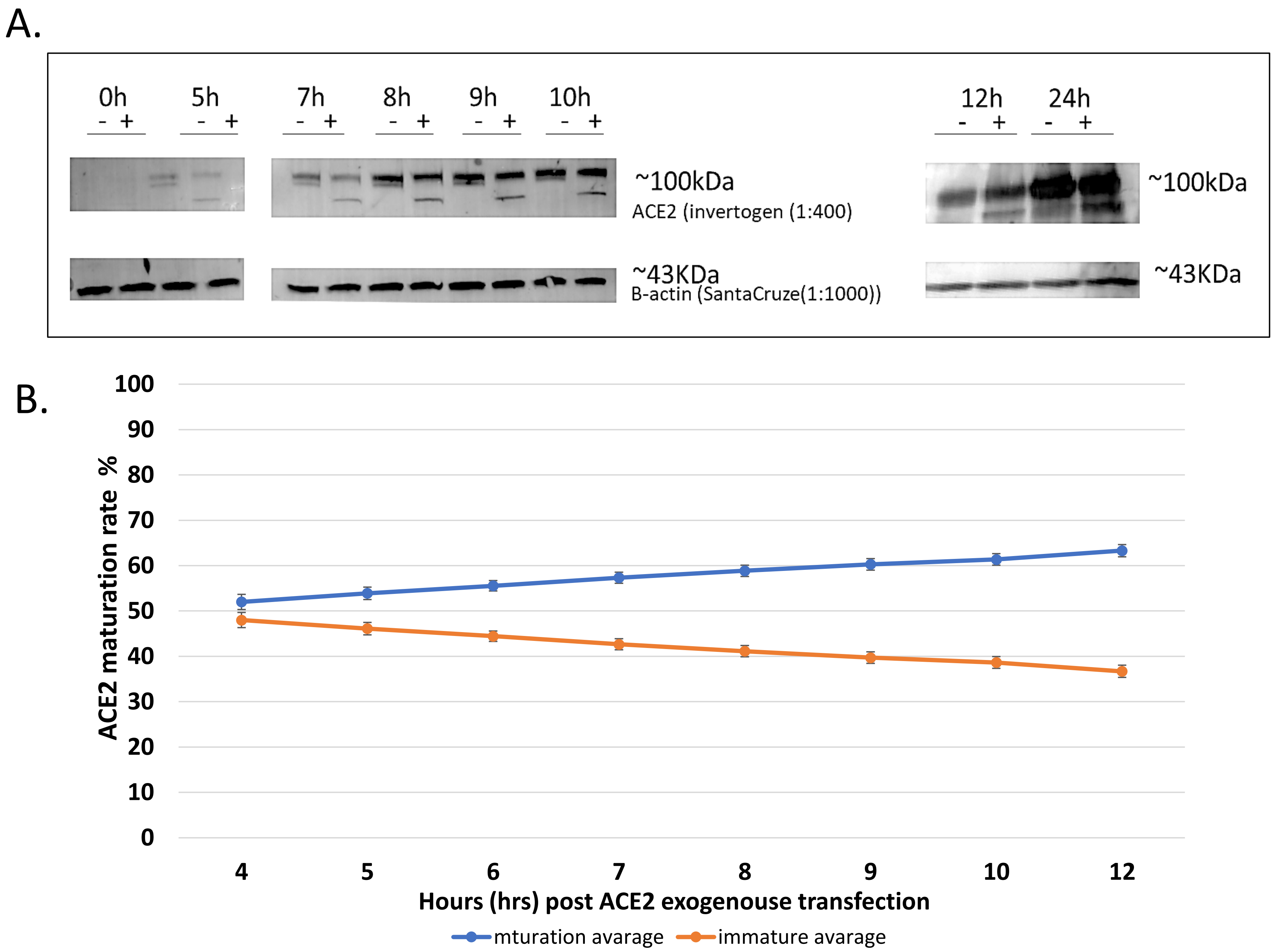
A.
0h
5h
7h
8h
9h
10h
12h
24h
- +
~100kDa
ACE2 (invertogen (1:400)
~43KDa
B-actin (SantaCruze(1:1000))
B.
ACE2 maturation rate %
100
90
80
70
60
50
40
30
20
10
0
4
5
6
7
8
9
10
12
Hours (hrs) post ACE2 exogenouse transfection
mturation avarage
immature avarage

Blots for Figure1

(not for publication)

- Protein ladder used is: precision plus protein ladder.

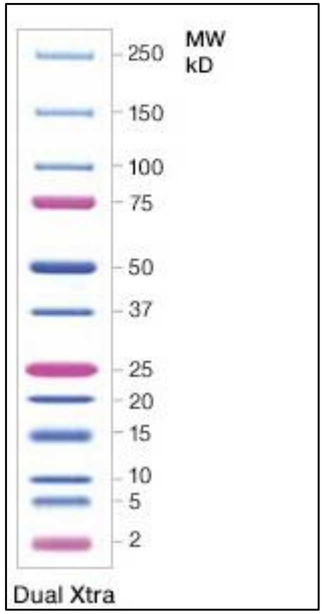

Anti-ACE2 Invitrogen (120kDa)

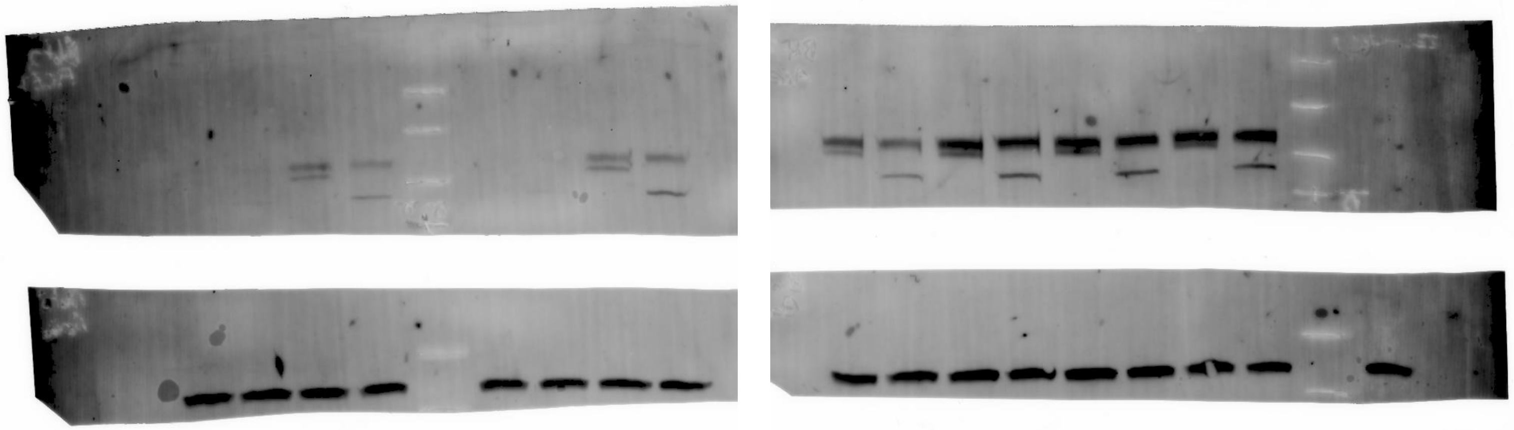

Anti-B actin Santacruz (~43kDa)

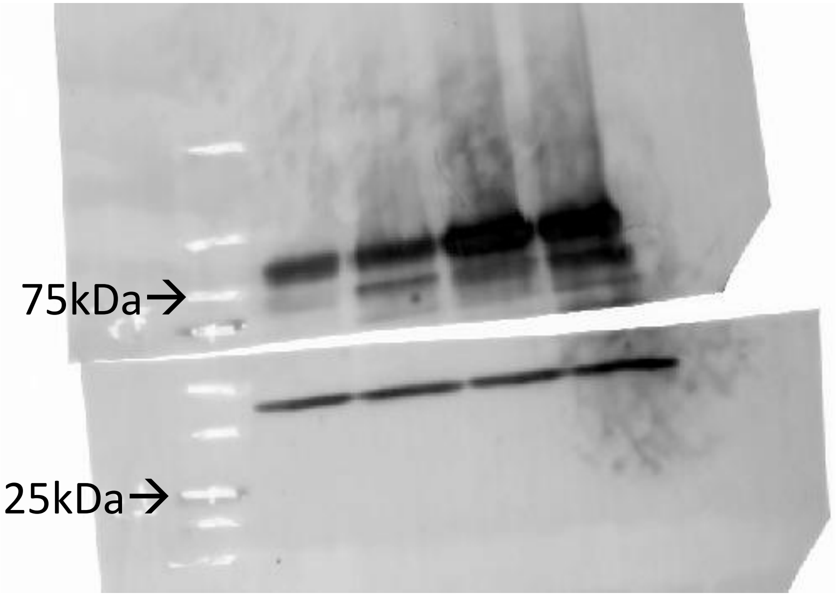

Some repeats done for Figure1

(not for publication)

0h 4h 6h 8h 12h 24h
\- + - + - + - + - + - +

100
75

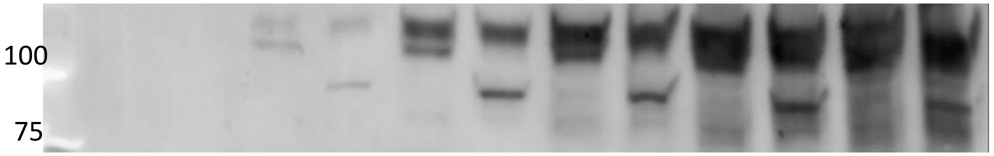

~100kDa
ACE2 (Invitrogen (1:400))

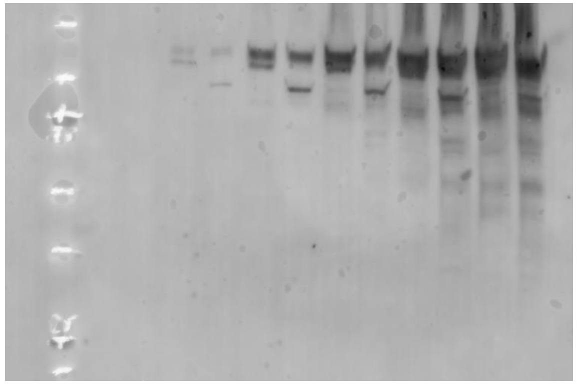

Blots developed with ECL plus developing reagent @450 nm wavelength using the Typhoon machine.

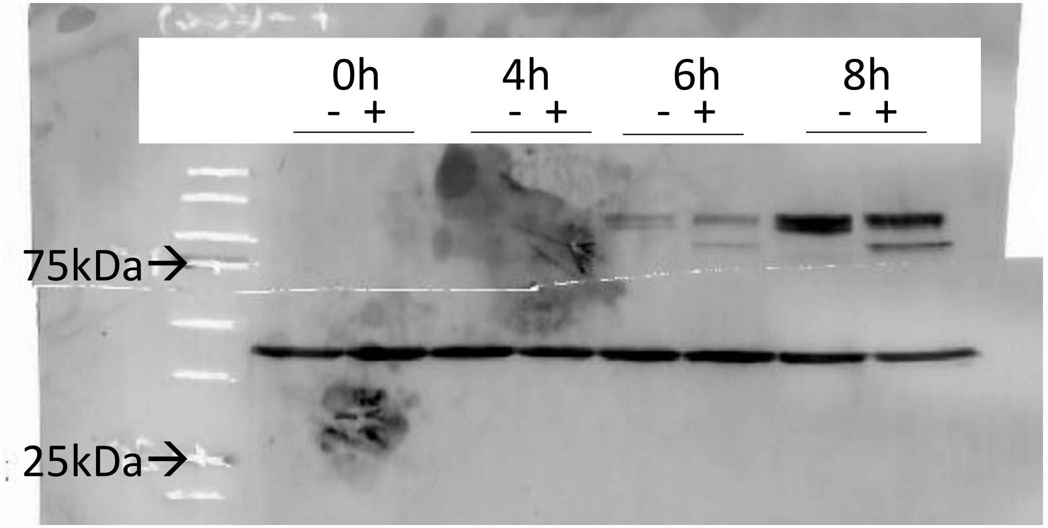

← Anti-ACE2 Invitrogen (120kDa)

← Anti-B actin Santacruz (~43kDa)

Figure3

Positive control

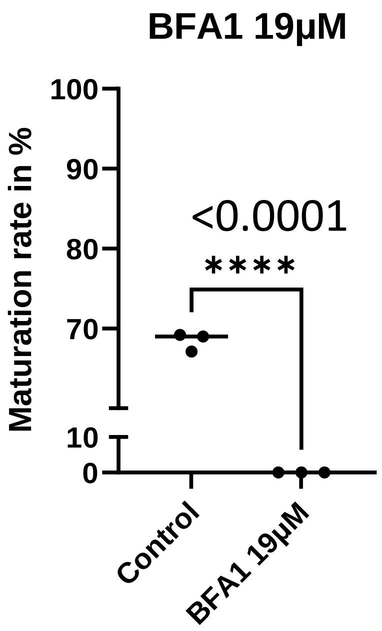

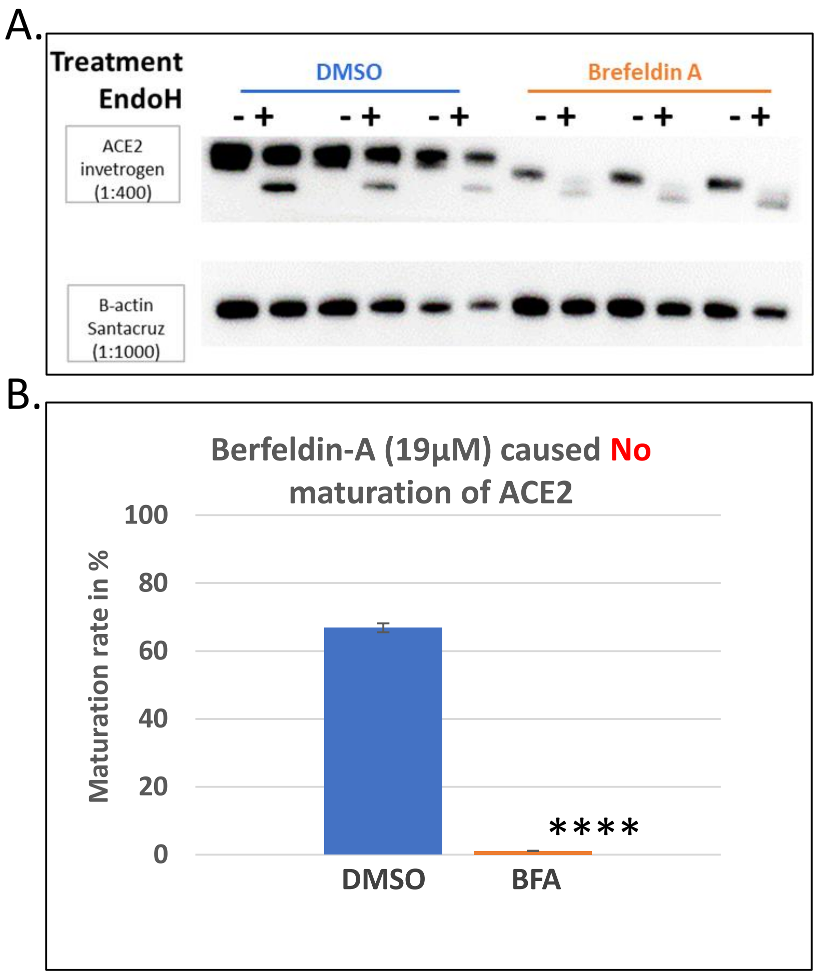

(not for publication)

Blots for Figure3 - Protein ladder used is: precision plus protein ladder.

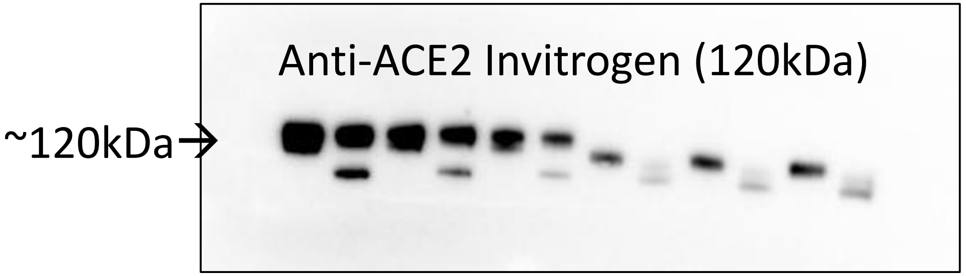

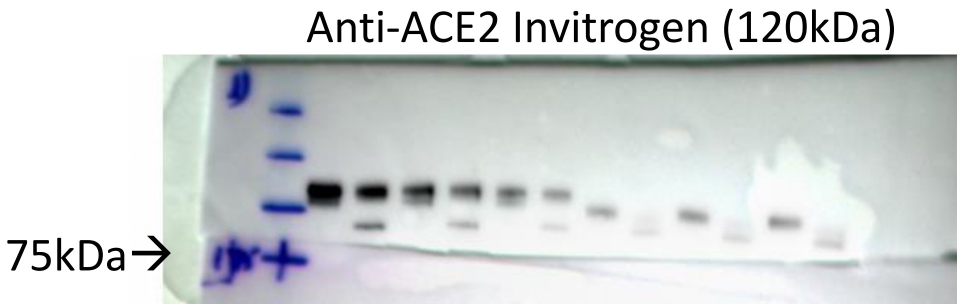

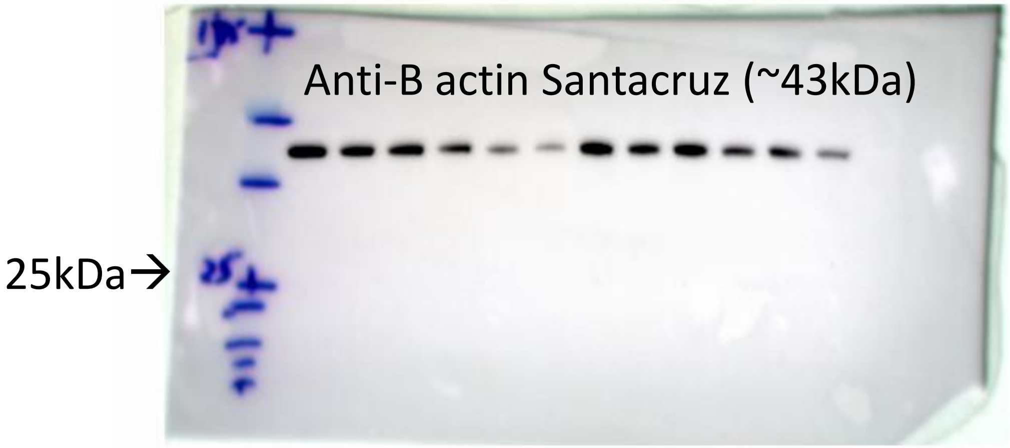

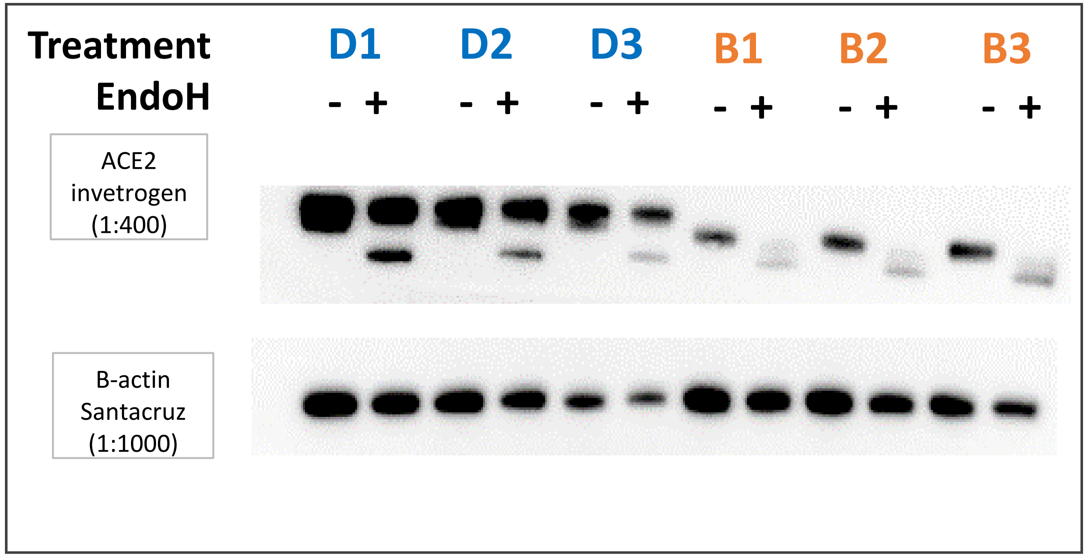

Figure4

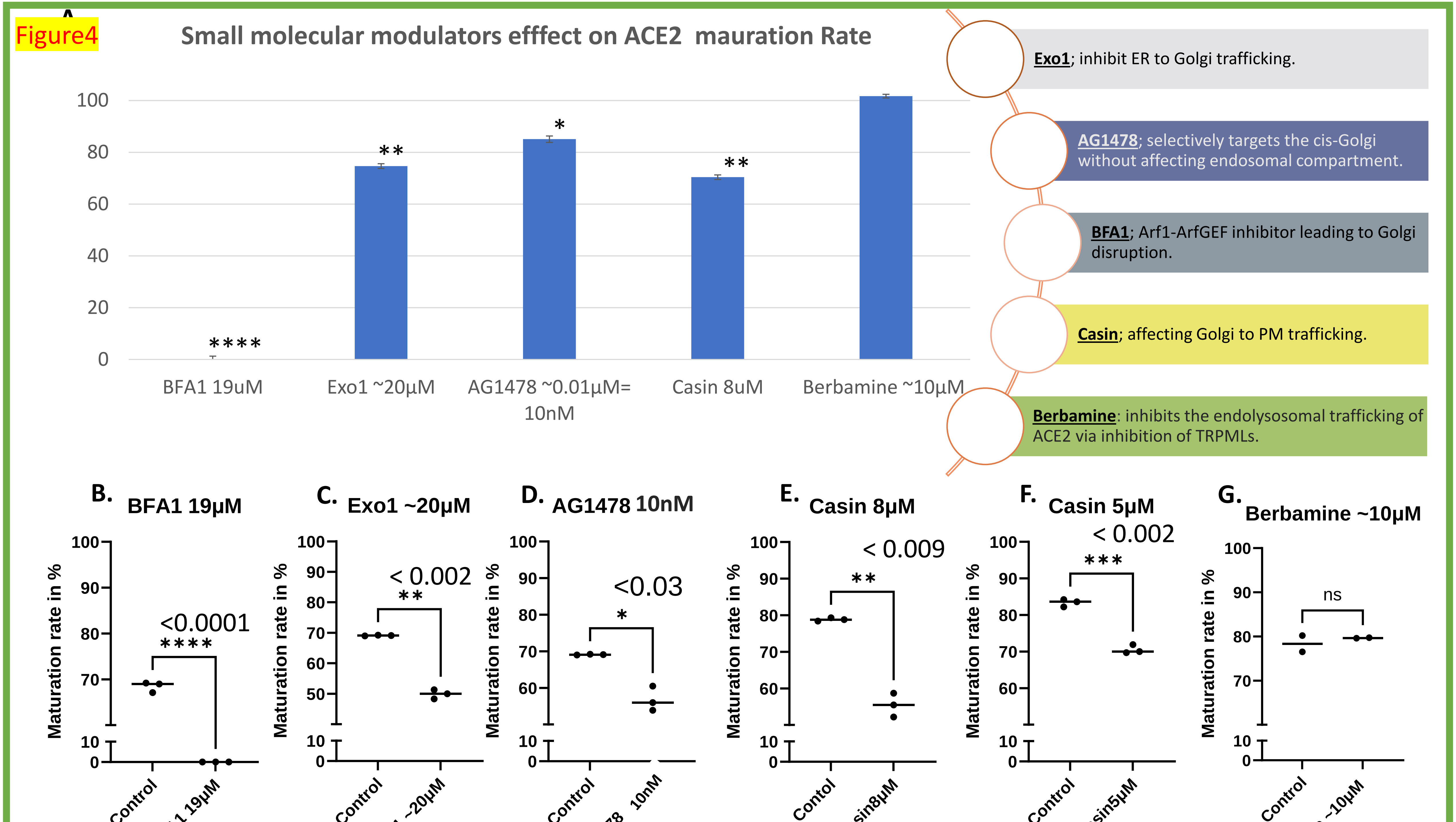

A
Small molecular modulators efffect on ACE2 mauration Rate
100
80
60
40
20
0
****
**
*
**
BFA1 19uM
Exo1 ~20µM
AG1478 ~0.01µM= 10nM
Casin 8uM
Berbamine ~10µM
Exo1; inhibit ER to Golgi trafficking.
AG1478; selectively targets the cis-Golgi without affecting endosomal compartment.
BFA1; Arf1-ArfGEF inhibitor leading to Golgi disruption.
Casin; affecting Golgi to PM trafficking.
Berbamine: inhibits the endolysosomal trafficking of ACE2 via inhibition of TRPMLs.
B. BFA1 19µM
<0.0001
****
C. Exo1 ~20µM
< 0.002
**
D. AG1478 10nM
<0.03
*
E. Casin 8µM
< 0.009
**
F. Casin 5µM
< 0.002
***
G. Berbamine ~10µM
ns
Maturation rate in %
Control
Contol

(not for publication)

Blots for Figure4; (1/3)

- Protein ladder used is: precision plus protein ladder.

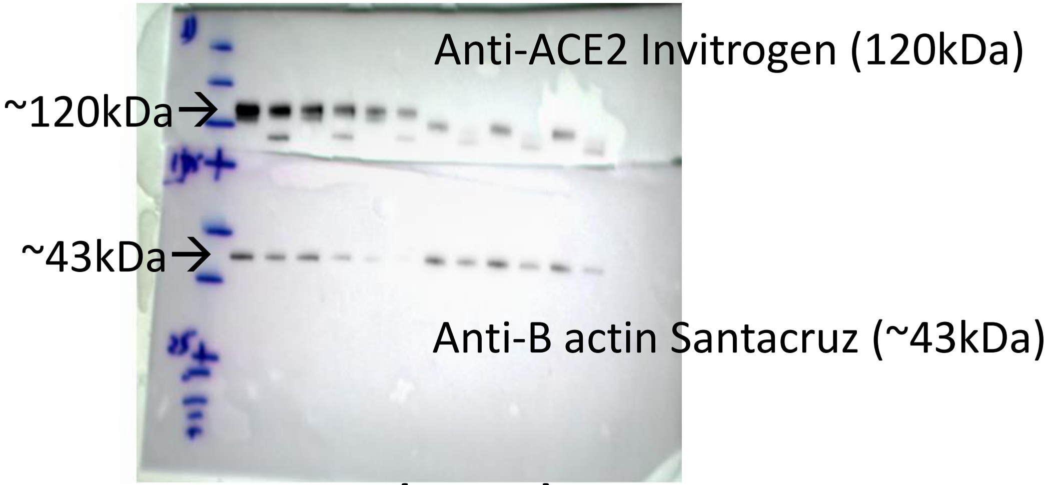

BFA (19uM)

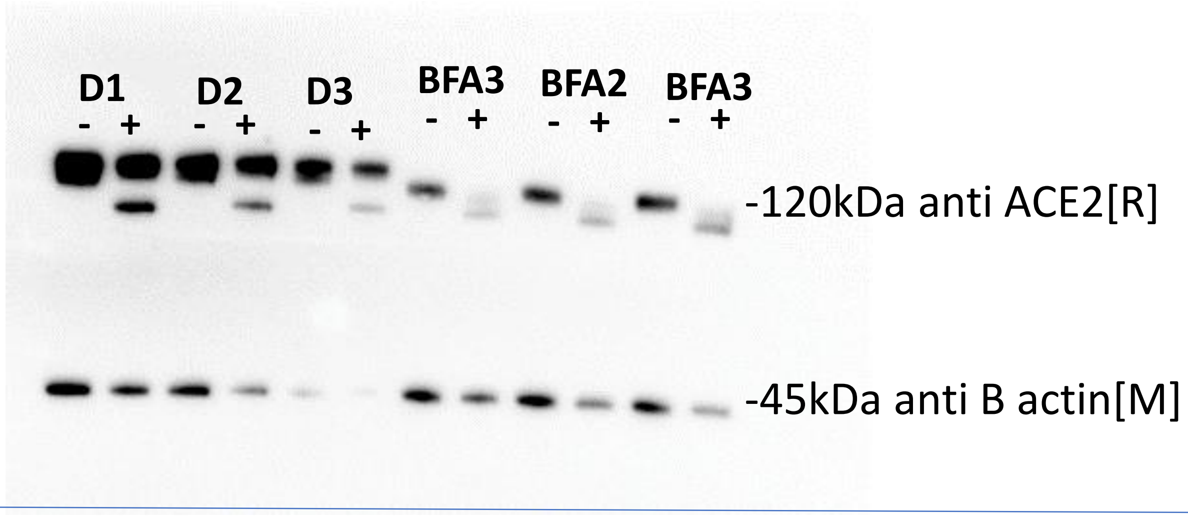

Ladder image is super imposed on raw blot, image taken from developing software.

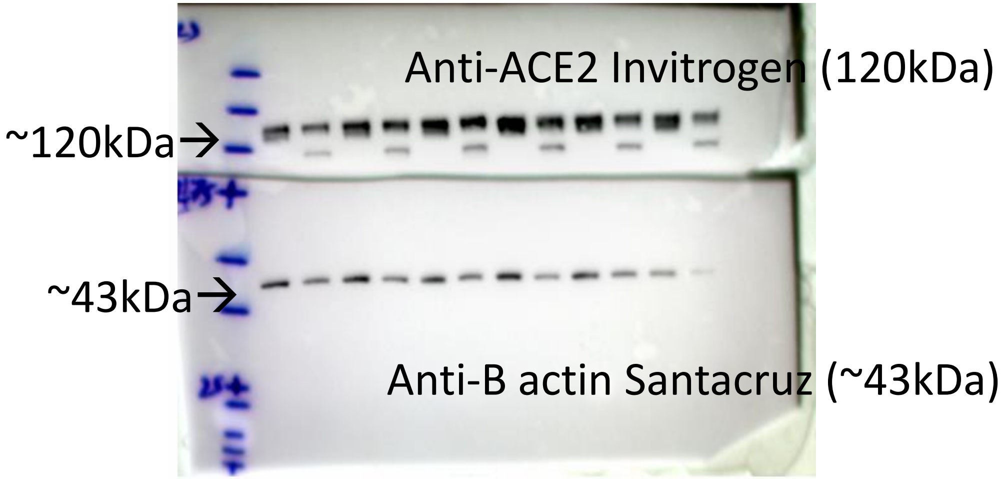

AG1478 (10nM) and Exo1 (20uM)

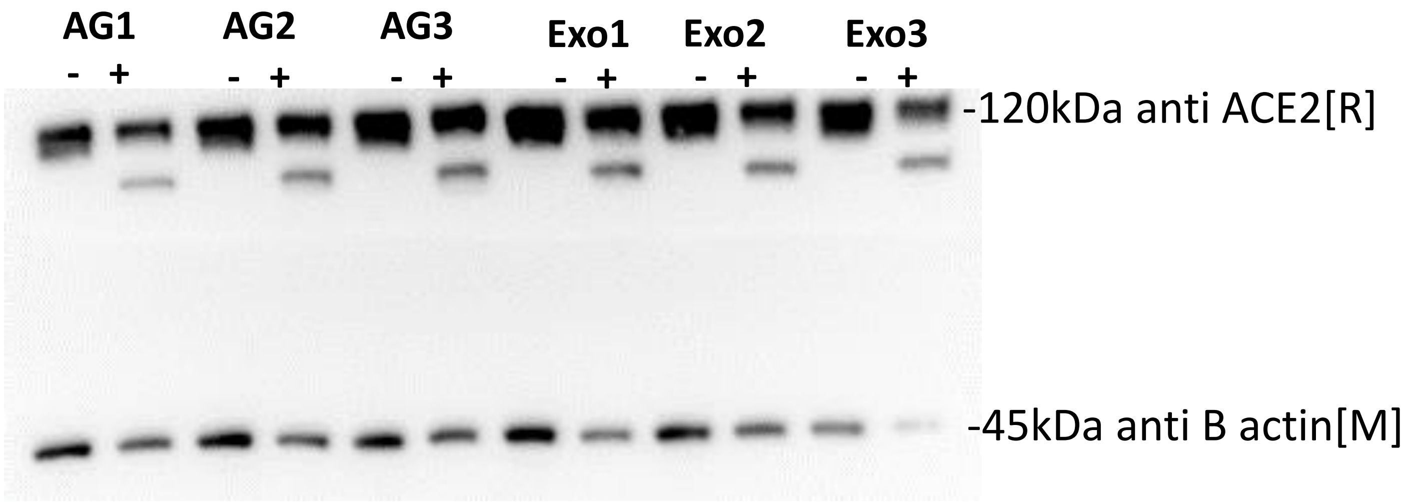

(not for publication)

Blots for Figure4; (2/3)

- Protein ladder used is: precision plus protein ladder.

Anti-ACE2 Invitrogen (120kDa)

~120kDa→

~43kDa→

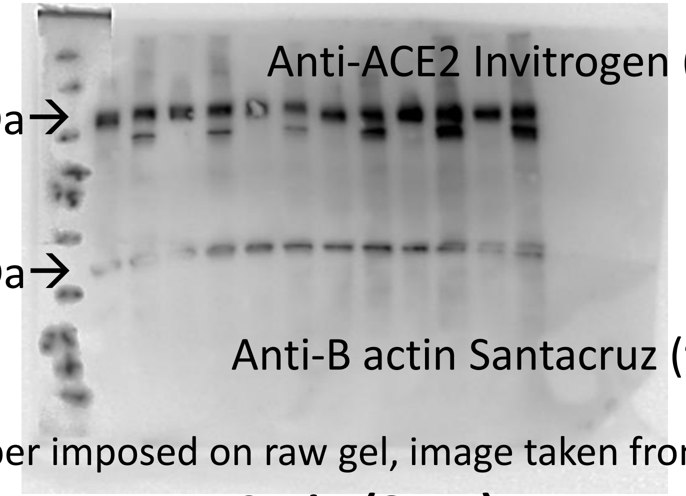

Anti-B actin Santacruz (~43kDa)

Only ladder is super imposed on raw gel, image taken from developing software.

Casin (8uM)

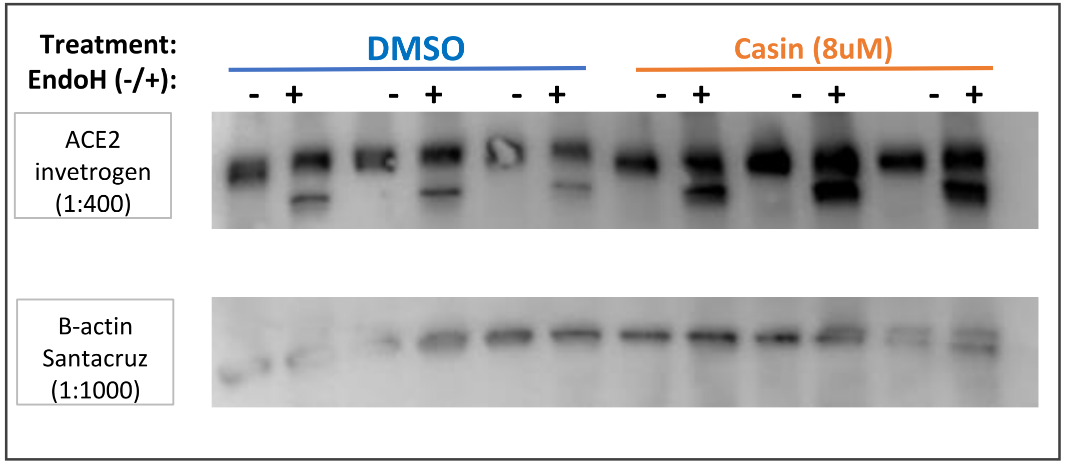

Anti-ACE2 Invitrogen (120kDa)

~120kDa→

~43kDa→

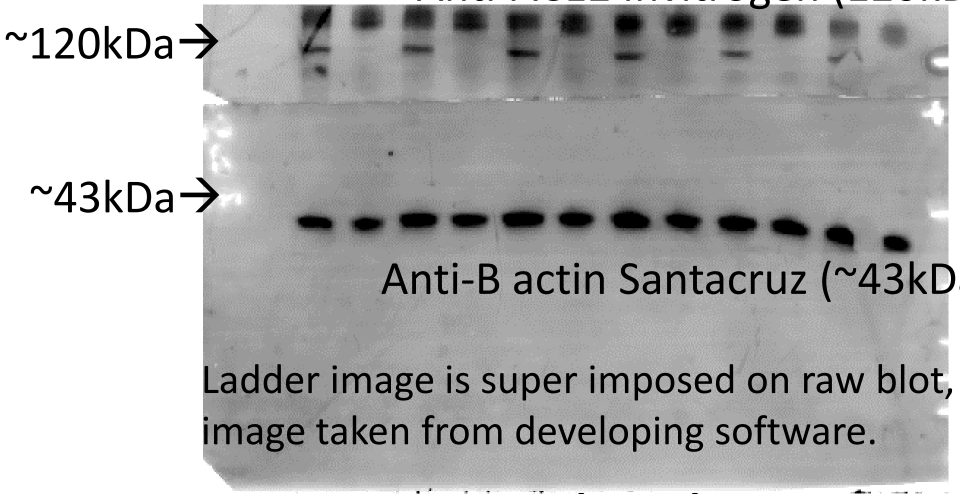

Anti-B actin Santacruz (~43kDa)

Ladder image is super imposed on raw blot, image taken from developing software.

Casin (5uM)

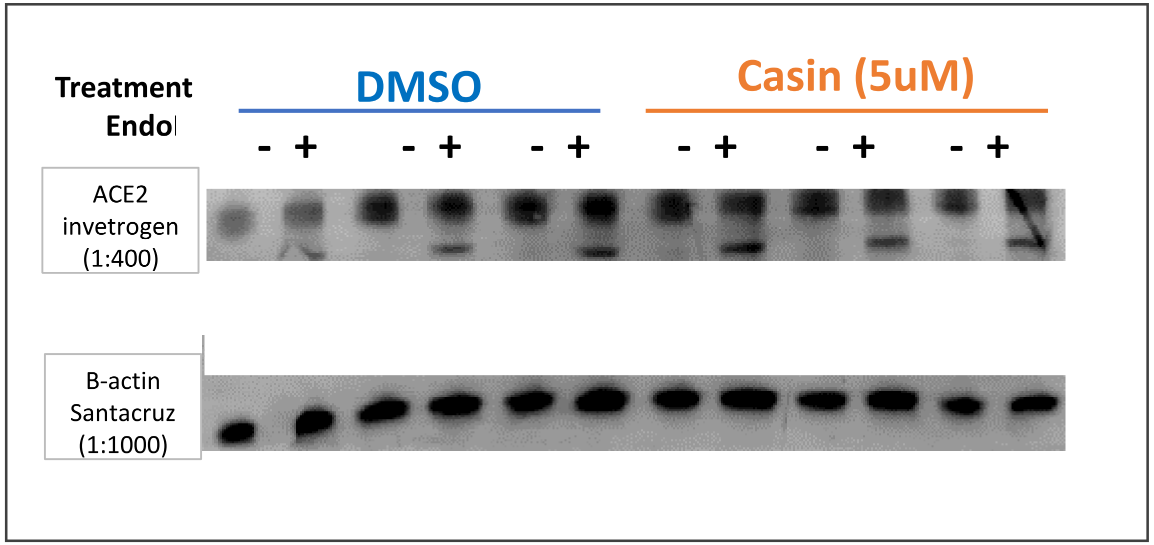

(not for publication)

Blots for Figure4; (3/3)

- Protein ladder used is: precision plus protein ladder.

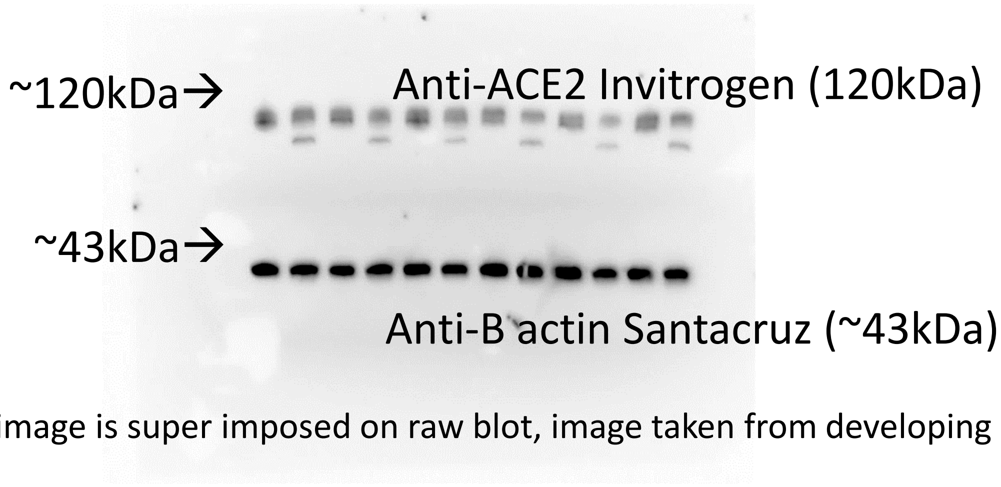

Ladder image is super imposed on raw blot, image taken from developing software.

Berbamine (10uM)

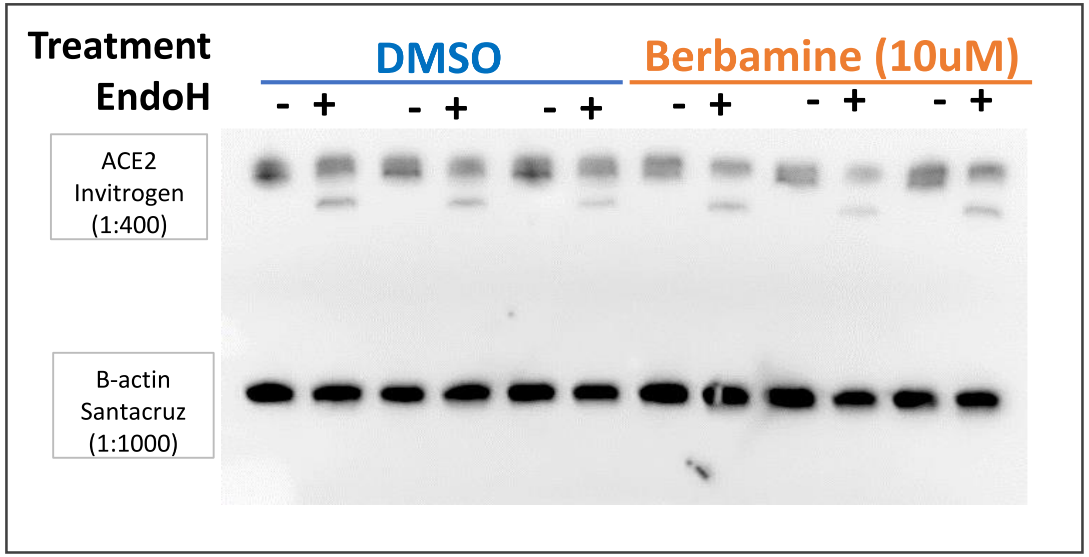

Figure5

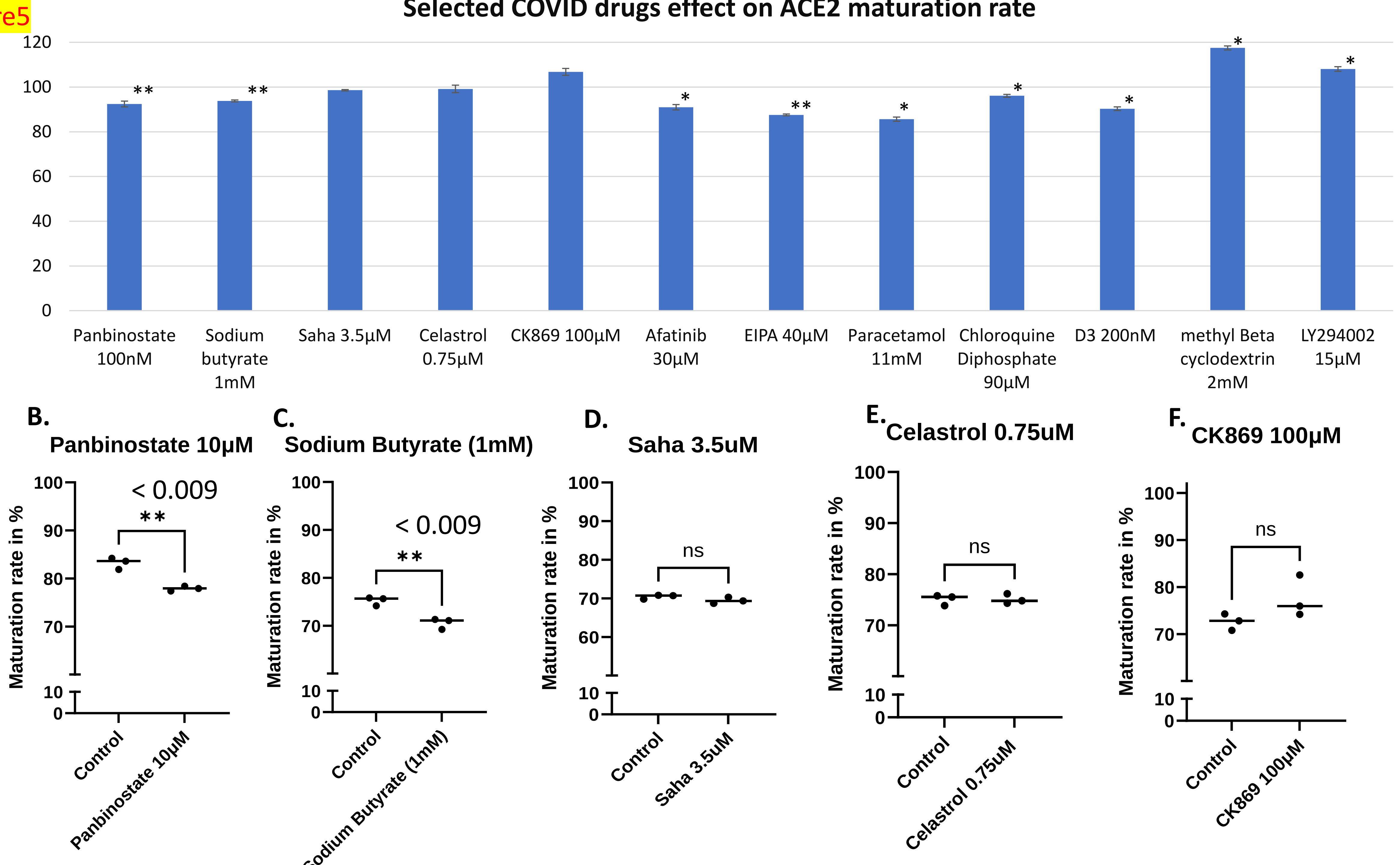

Selected COVID drugs effect on ACE2 maturation rate
120
100
80
60
40
20
0
**
**
*
**
*
*
*
*
*
Panbinostate 100nM
Sodium butyrate 1mM
Saha 3.5µM
Celastrol 0.75µM
CK869 100µM
Afatinib 30µM
EIPA 40µM
Paracetamol 11mM
Chloroquine Diphosphate 90µM
D3 200nM
methyl Beta cyclodextrin 2mM
LY294002 15µM
B.
Panbinostate 10µM
Maturation rate in %
< 0.009
**
Control
Panbinostate 10µM
C.
Sodium Butyrate (1mM)
Maturation rate in %
< 0.009
**
Control
Sodium Butyrate (1mM)
D.
Saha 3.5uM
Maturation rate in %
ns
Control
Saha 3.5uM
E.
Celastrol 0.75uM
Maturation rate in %
ns
Control
Celastrol 0.75uM
F.
CK869 100µM
Maturation rate in %
ns
Control
CK869 100µM

(not for publication)

Blots for Figure5; (1/6)

- Protein ladder used is: precision plus protein ladder.

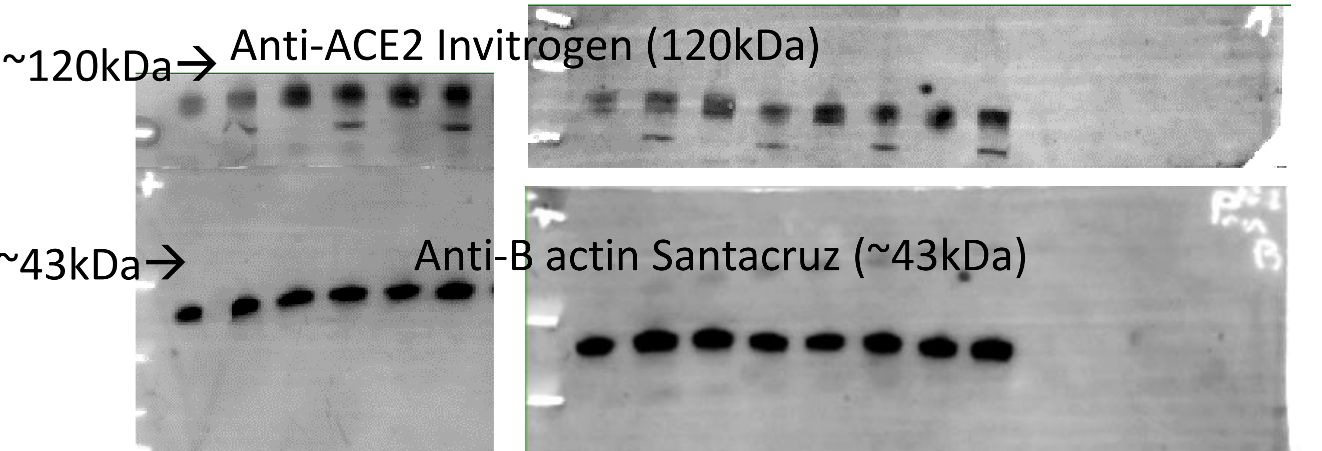

Ladder image is super imposed on raw blots, images taken from developing software.

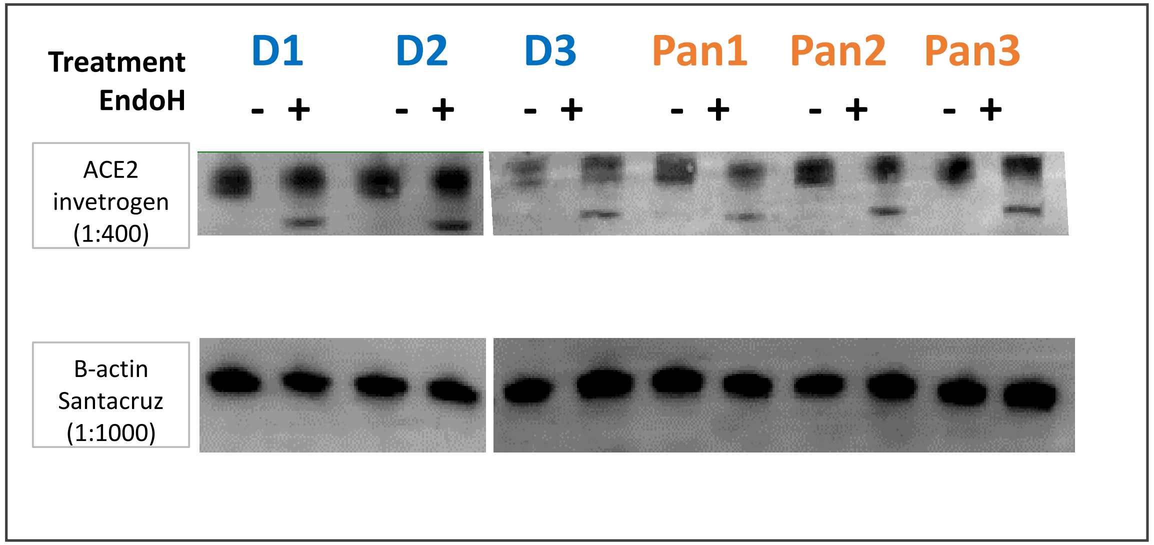

Panobinostat (10uM)

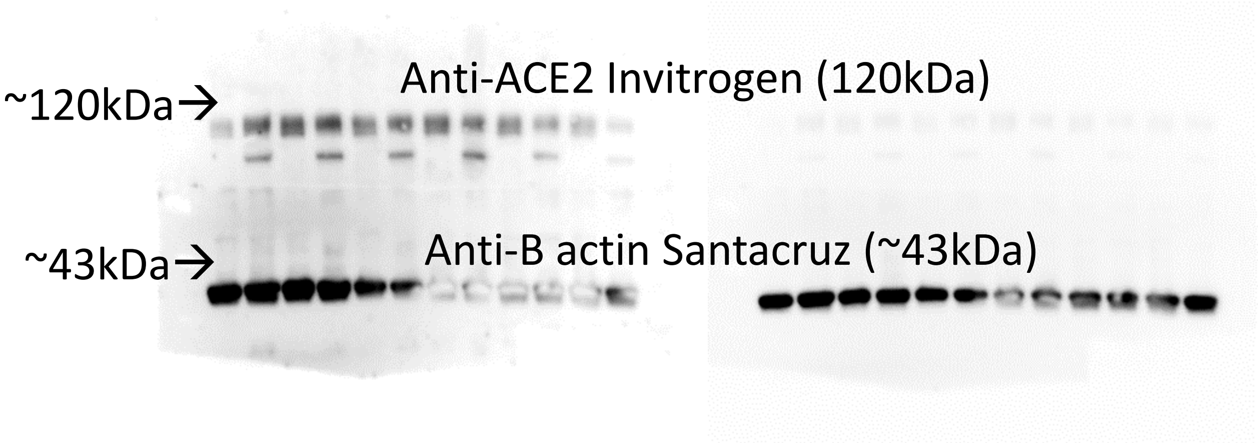

Ladder image is super imposed on raw blots, images taken from developing software.

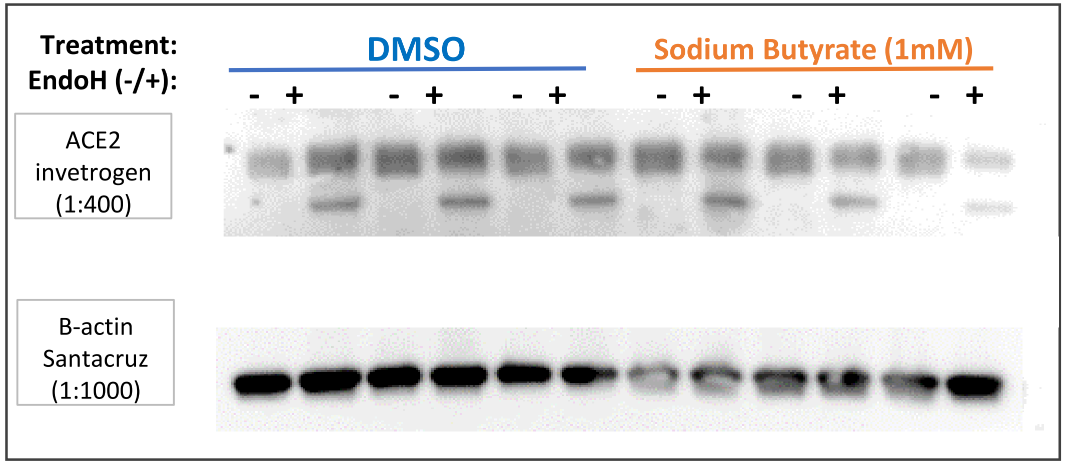

Sodium Butyrate (1mM)

(not for publication)

Blots for Figure5; (2/6)

- Protein ladder used is: precision plus protein ladder.

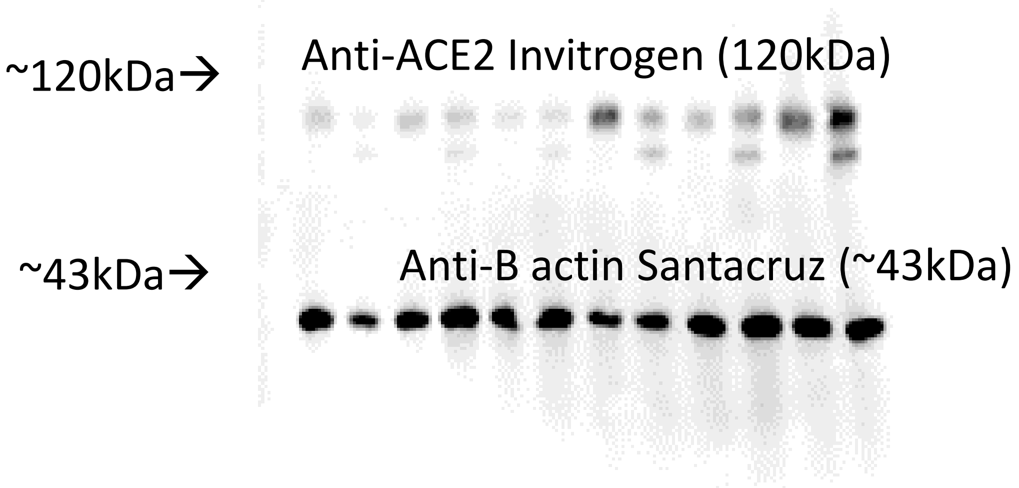

SAHA (3.5uM)

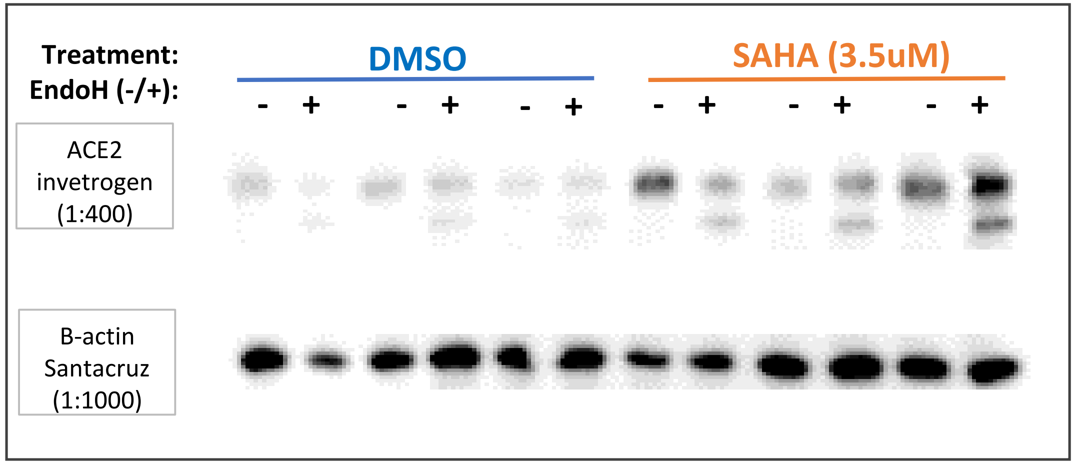

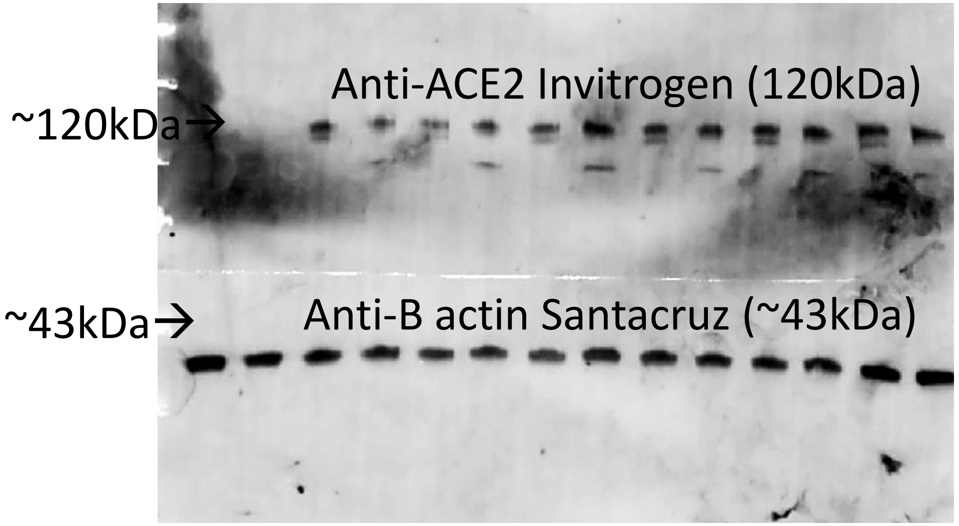

Celastrol (0.75uM)

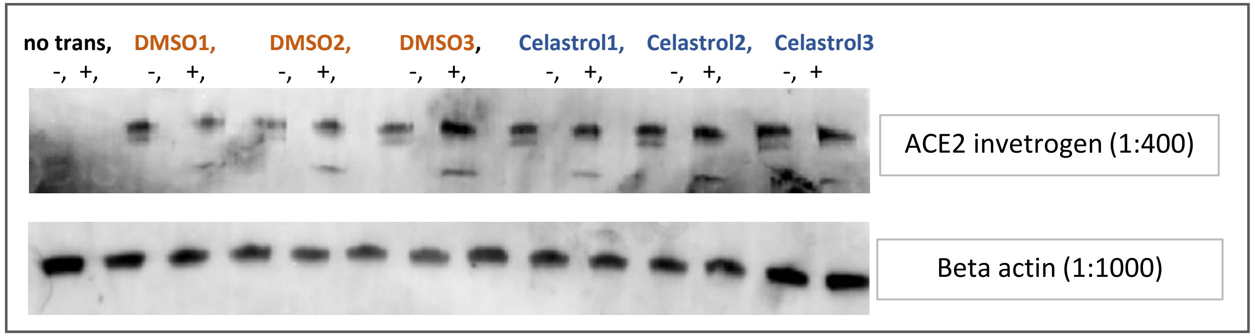

(not for publication)

Blots for Figure5; (3/6)

- Protein ladder used is: precision plus protein ladder.

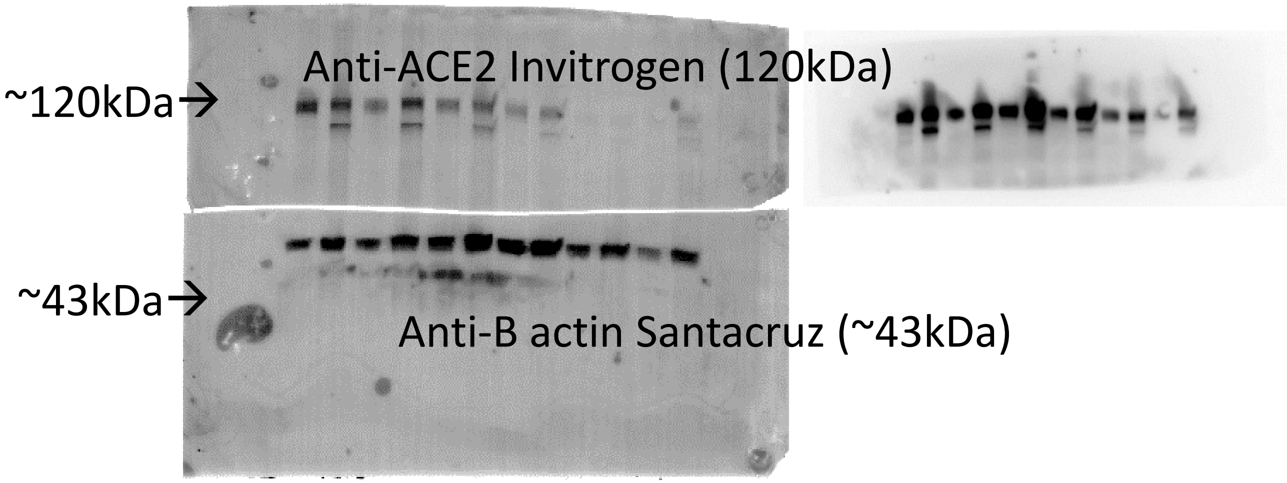

CK869 (100uM)

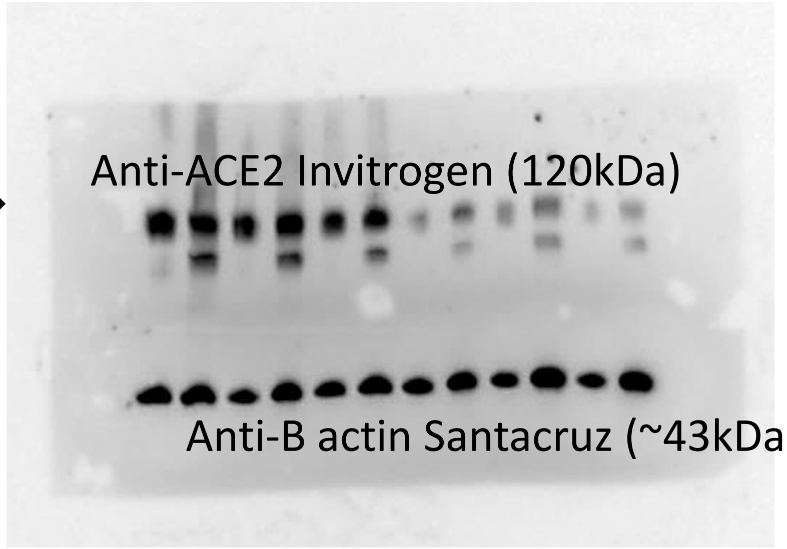

Afatinib (30uM)

(not for publication)

Blots for Figure5; (4/6)

- Protein ladder used is: precision plus protein ladder.

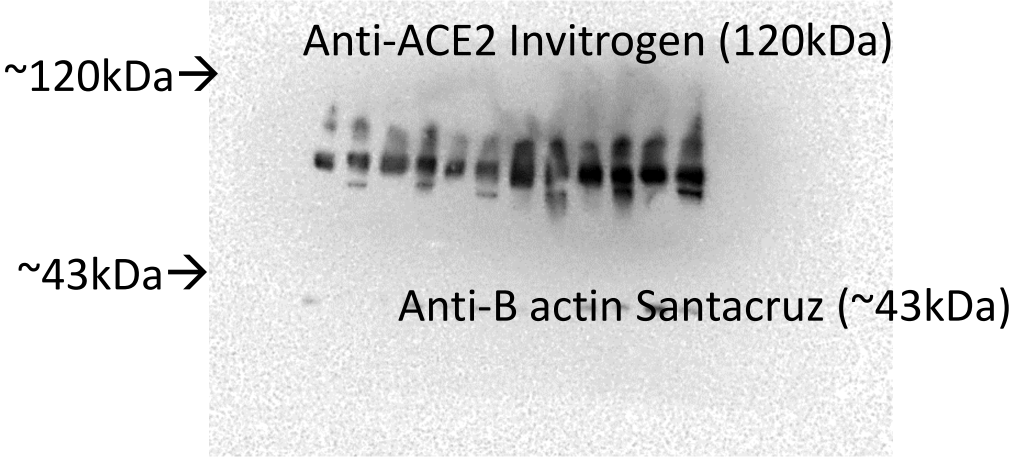

EIPA (40uM)

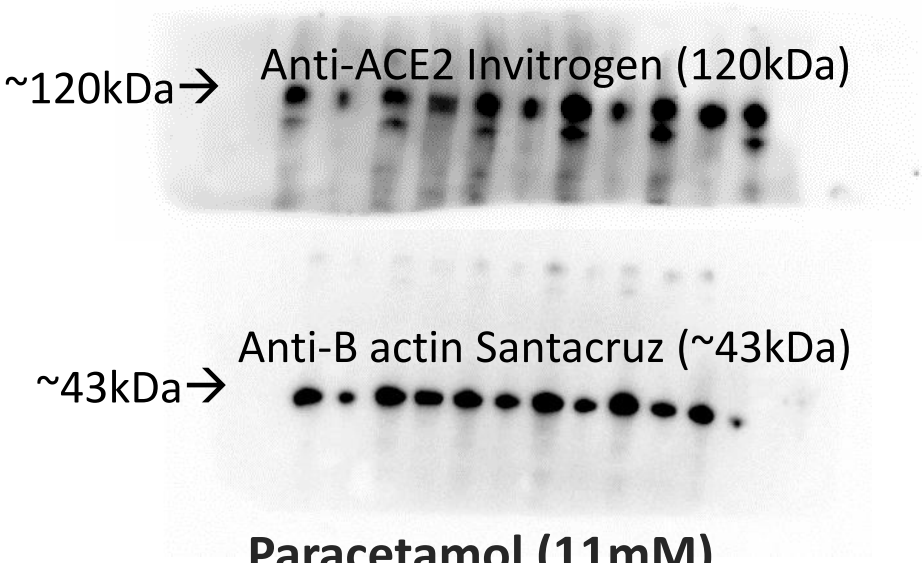

Paracetamol (11mM)

(not for publication)

Blots for Figure5; (5/6)

- Protein ladder used is: precision plus protein ladder.

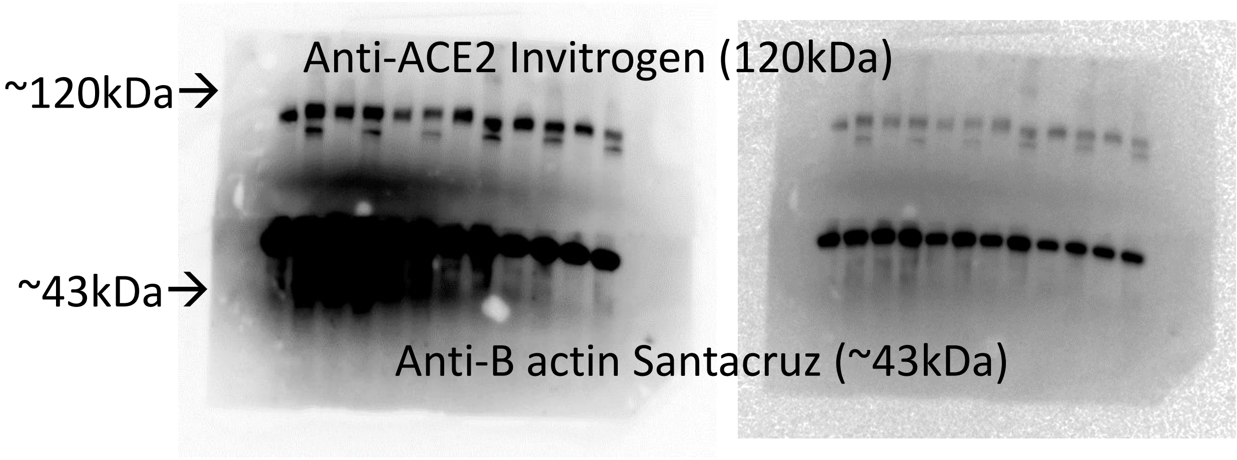

Chloroquine diphosphate (90uM)

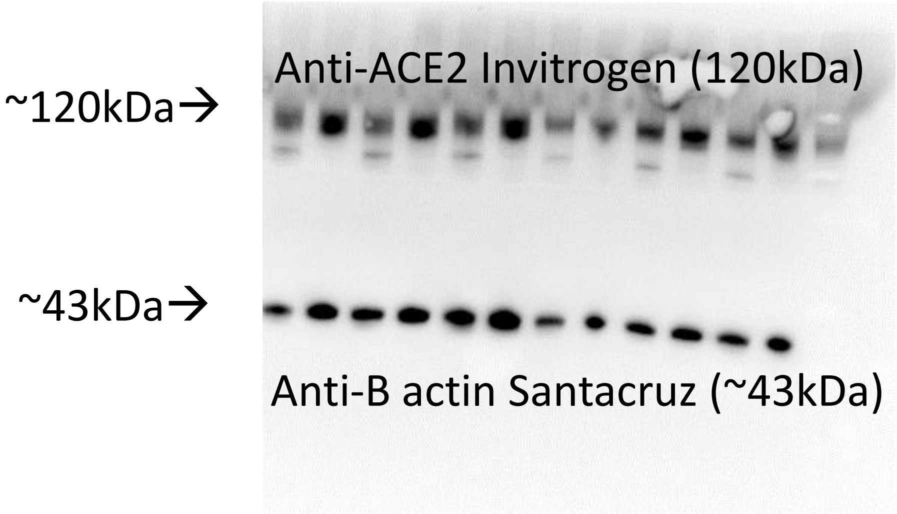

Methyl Beta Cyclodextrin (2mM)

(not for publication)

Blots for Figure5; (6/6)

- Protein ladder used is: precision plus protein ladder.

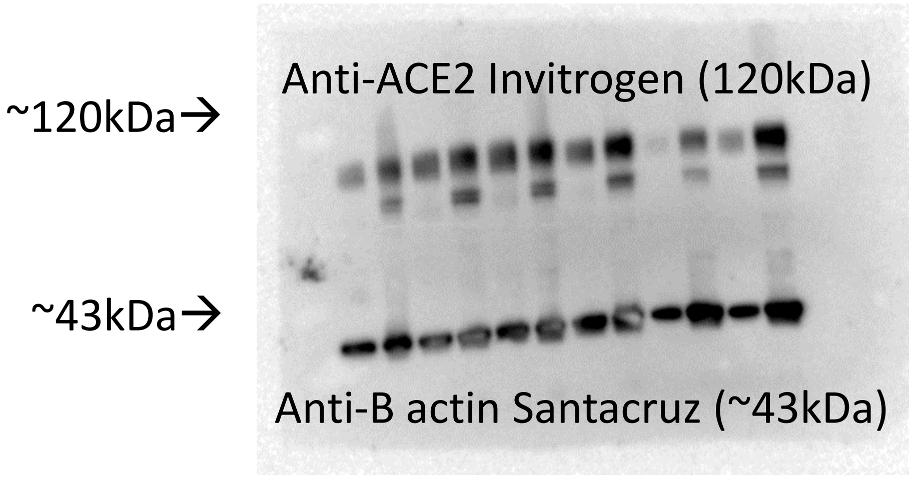

LY294002 (15µM)

Figure6

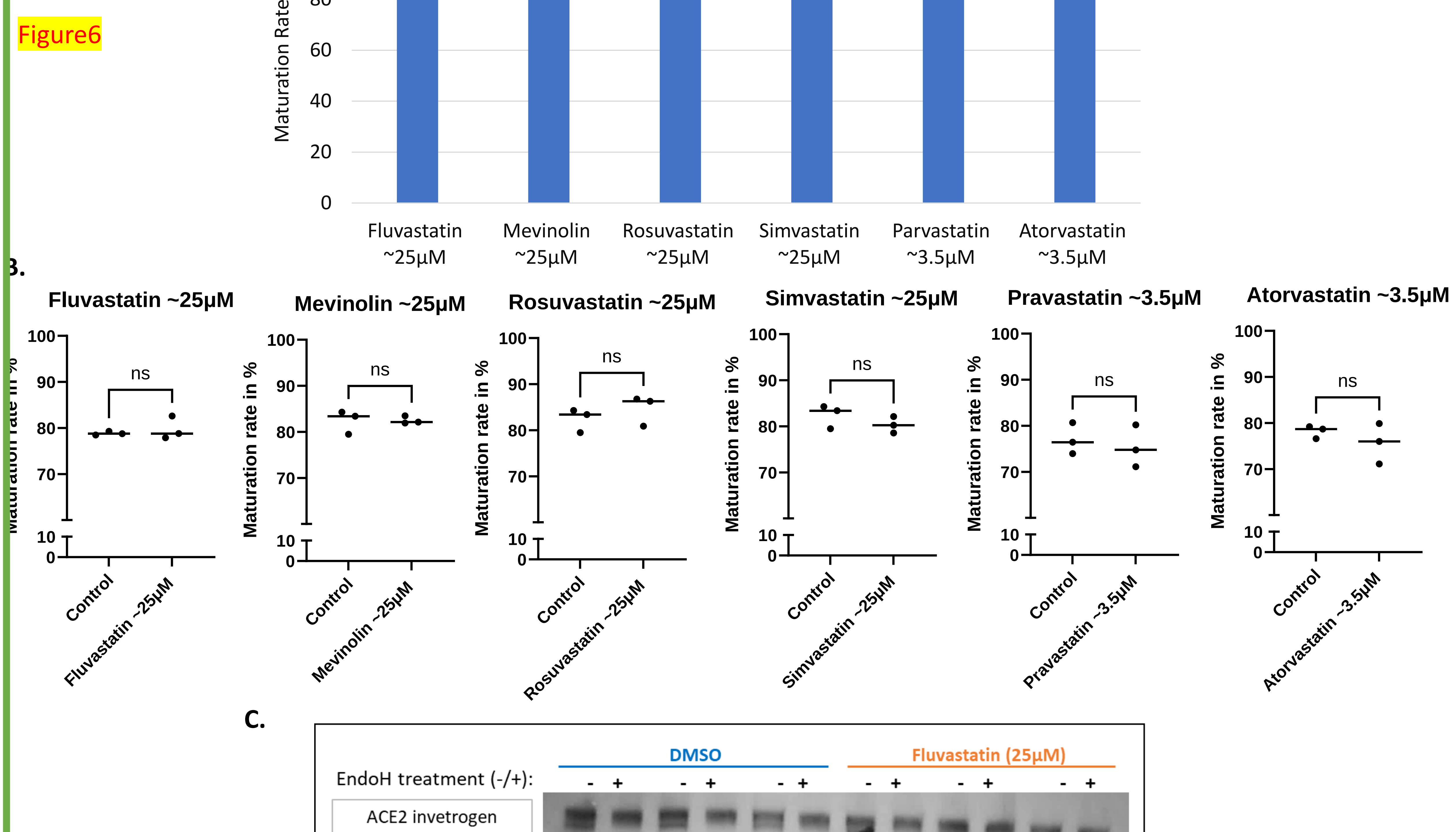

Maturation Rate
80
60
40
20
0
Fluvastatin ~25µM
Mevinolin ~25µM
Rosuvastatin ~25µM
Simvastatin ~25µM
Parvastatin ~3.5µM
Atorvastatin ~3.5µM
B.
Fluvastatin ~25µM
Mevinolin ~25µM
Rosuvastatin ~25µM
Simvastatin ~25µM
Pravastatin ~3.5µM
Atorvastatin ~3.5µM
Maturation rate in %
100
90
80
70
10
0
ns
Control
Fluvastatin ~25µM
Mevinolin ~25µM
Rosuvastatin ~25µM
Simvastatin ~25µM
Pravastatin ~3.5µM
Atorvastatin ~3.5µM
C.
DMSO
Fluvastatin (25µM)
EndoH treatment (-/+):
- + - + - + - + - + - +
ACE2 invetrogen

(not for publication)

Blots for Figure6; (1/3)

- Protein ladder used is: precision plus protein ladder.

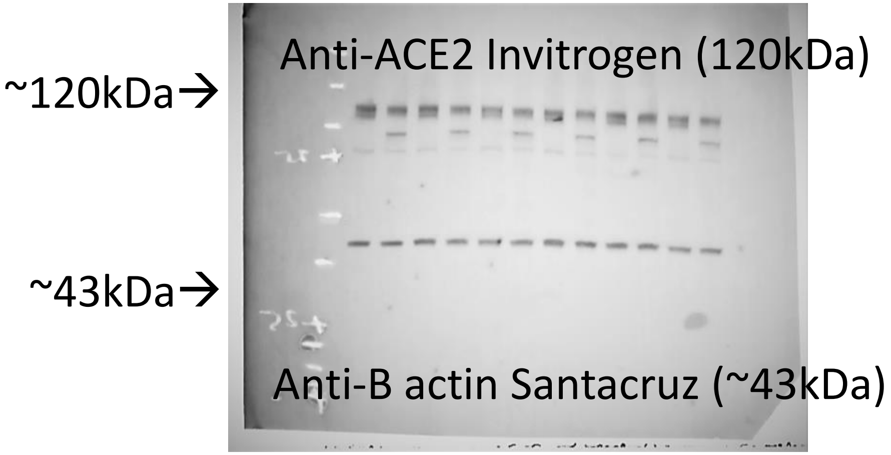

Fluvastatin (25µM)

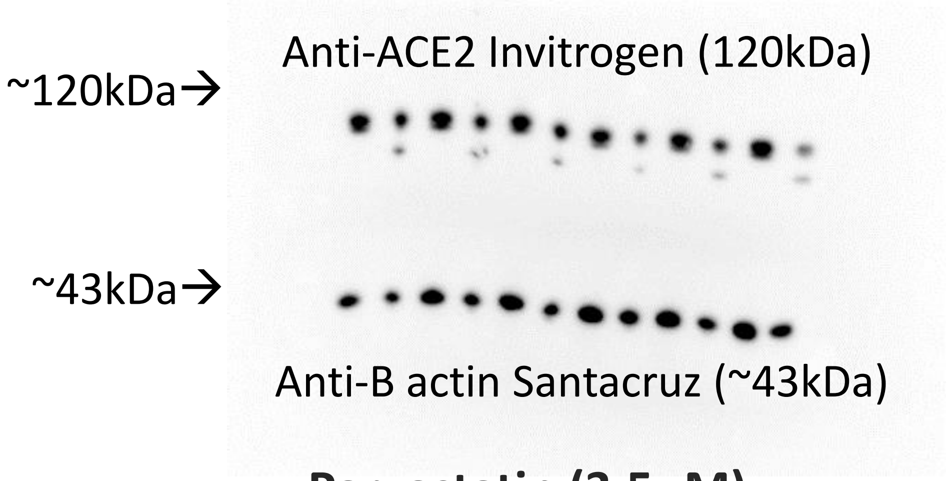

Parvastatin (3.5µM)

(not for publication)

Blots for Figure6; (2/3)

- Protein ladder used is: precision plus protein ladder.

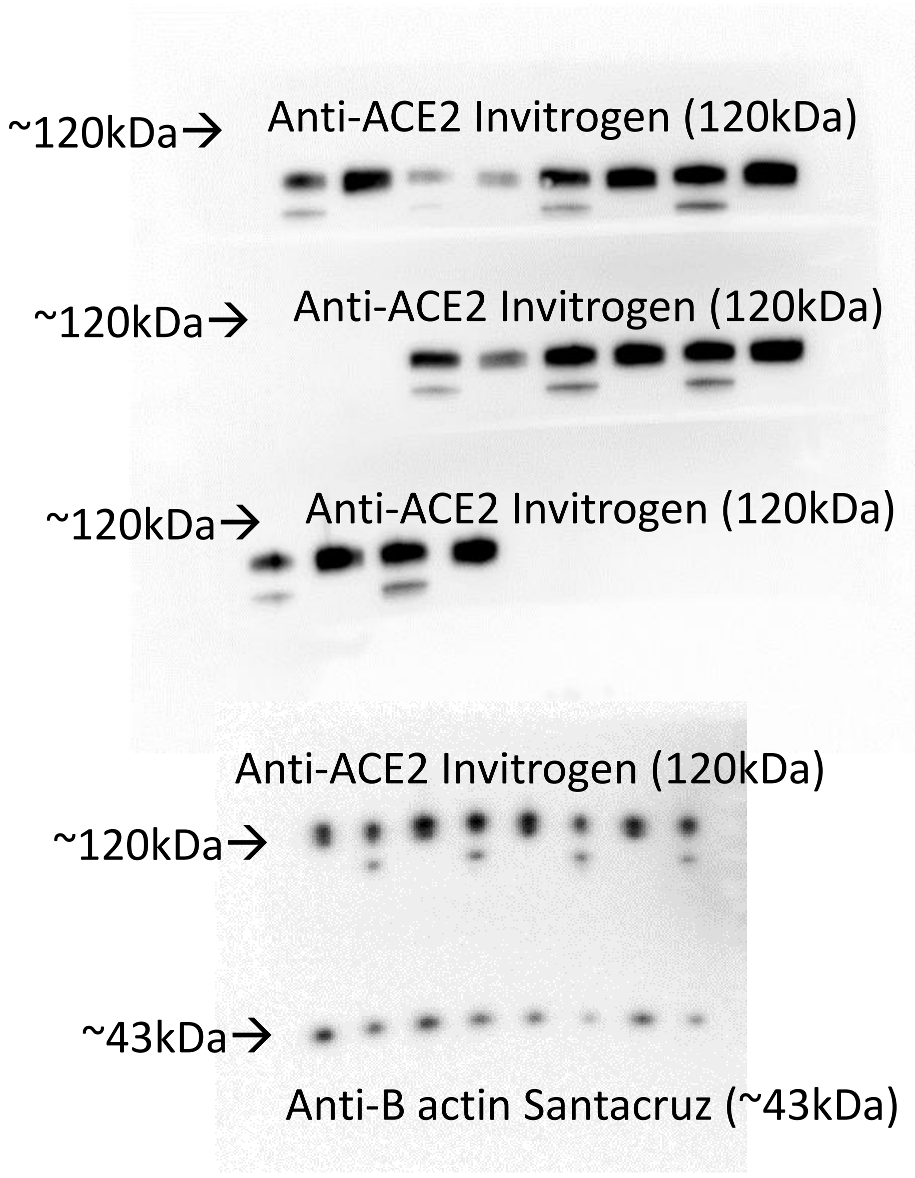

(not for publication)

Blots for Figure6; (3/3)

- Protein ladder used is: precision plus protein ladder.

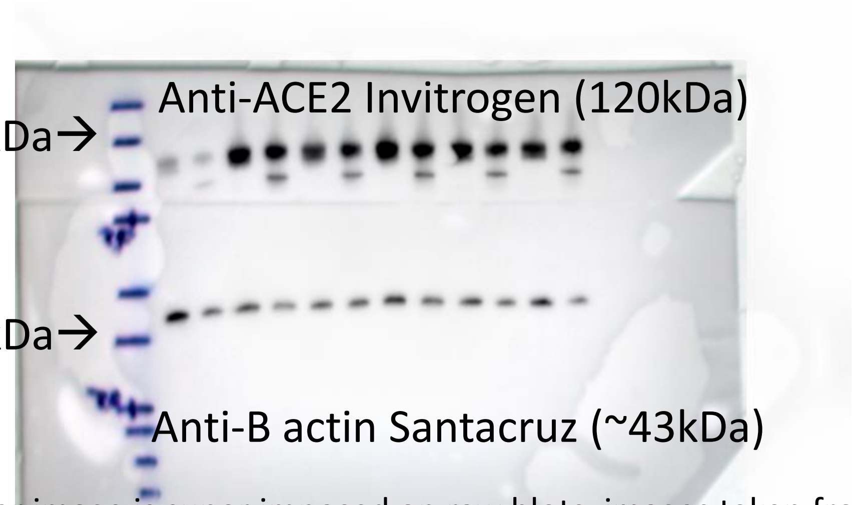

Ladder image is super imposed on raw blots, images taken from developing software.

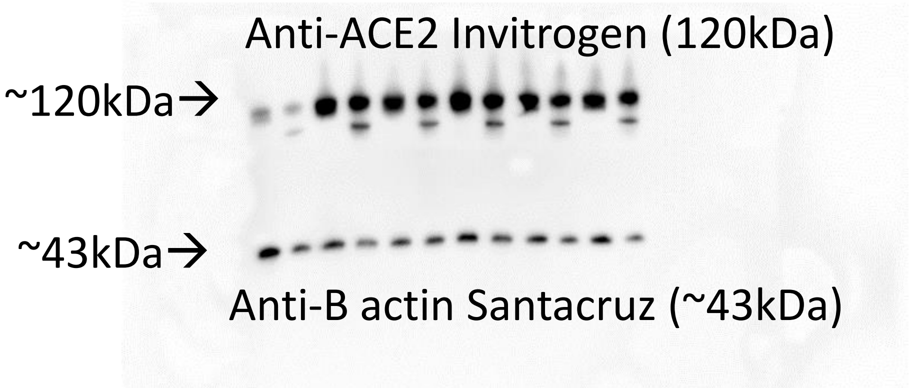

Raw blot without ladder superimposed on it.

Atorvastatin (~3.5µM)
