# Supplementary material for: The Evaluation of Drugs as Potential Modulators of the Trafficking and Maturation of ACE2, the SARS-CoV-2 Receptor
Source: Biomolecules. 2024 Jun 27;14(7):764. doi: 10.3390/biom14070764 (PMC11274373; doi:10.3390/biom14070764)
Supplement: Supplementary file 1 [file biomolecules-14-00764-s001.zip › biomolecules-3041240-supplementary.pdf]

Supplementary material:

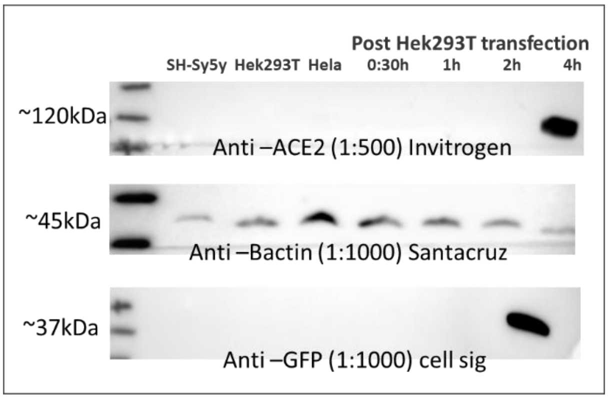

Supplementary-Figure S1: Western blot showing the none transfected Cell lines for naturally expressed ACE2, in addition to Hek293T post ACE2 transfection at different time points. Transfection agent used is FUGENE-HD. ACE2 construct used is OriGene RC208442. B-actin as loading control. GFP as transfection control.

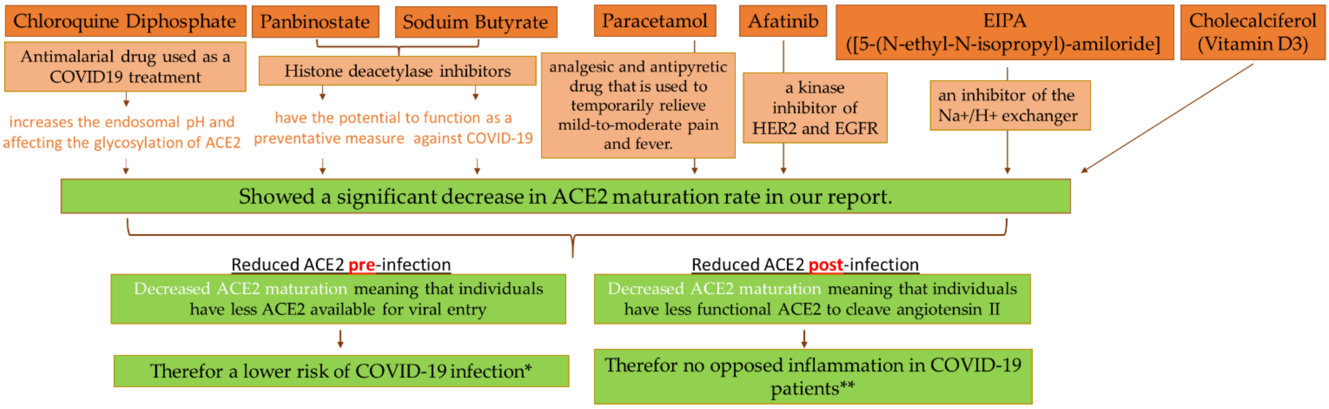

Supplementary-Figure S2; Diagram of the screened drugs that caused significant reduction in ACE2 maturation. The postulated mechanism of action of the tested molecules showing reduced ACE2 maturation. Hypothesized mechanism of action of small molecules, showing modest reduction in ACE2 maturation. *Decreased ACE2 protein expression associated with a lower risk of COVID-19 infection [1]. **Less functional ACE2 ready for angiotensin II cleavage, has been reported to cause acute pulmonary inflammation and coagulation, these medical complications arise in response to enhanced and unopposed angiotensin II detrimental effects [2].

Supplementary-Table S1: Table of the literature citations that we have used to select the starting points for our optimization of the screening. Prior to deciding on the concentration of each of the tested drugs, we carried out literature search on the tolerable concentration and in most cases tested multiple concentrations around what was found in the literature.

#	Drug	Reference
1	Afatinib	[3]
2	AG1478	[4]
3	Atorvastatin	[5]
4	Berbamine hydrochloride	[6]
5	BFA1	[7]
6	Casin	[3]
7	Celastrol	[8]
8	Chloroquine Diphosphate	[9]
9	Cholecalciferol (Vitamin D3)	[10]
10	CK869	[3]
11	EIPA	[3]
12	Exo1	[11]
13	Fluvastatin	[5]
14	LY294002	[3]
15	Methyl-β-cyclodextrin	[12]
16	Mevinolin (Lovastatin)	[5]
17	Panobinostat	[13]
18	Paracetamol	[14]
19	Pravastatin	[5]
20	Rosouvastatin	[5]
21	Smivastatin	[5]
22	Sodium Butyrate	[15]
23	Vorinostat (SAHA)	[16]

References:

[1] N. Santos Leal, Y. Yu, Y. Chen, G. Fedele, L. M. Martins, and K. Poulas, "Paracetamol Is Associated with a Lower Risk of COVID-19 Infection and Decreased ACE2 Protein Expression: A Retrospective Analysis," *COVID 2021, Vol. 1, Pages 218-229*, vol. 1, no. 1, pp. 218–229, Aug. 2021, doi: 10.3390/COVID1010018.

[2] F. Angeli *et al.*, "The pivotal link between ACE2 deficiency and SARS-CoV-2 infection: One year later," *Eur. J. Intern. Med.*, vol. 93, pp. 28–34, Nov. 2021, doi: 10.1016/J.EJIM.2021.09.007.

[3] Y. Endo *et al.*, "Identification of a pharmacological approach to reduce ACE2 expression and development of an in vitro COVID-19 viral entry model," *J. virus Erad.*, vol. 8, no. 4, Dec. 2022, doi: 10.1016/J.JVE.2022.100307.

[4] K. Q. Xie, L. M. Zhang, Y. Cao, J. Zhu, and L. Y. Feng, "Adenosine A 1 receptor-mediated transactivation of the EGF receptor produces a neuroprotective effect on cortical neurons in vitro," *Acta Pharmacol. Sin.*, vol. 30, no. 7, pp. 889–898, Jul. 2009, doi: 10.1038/APS.2009.80.

[5] F. J. Zapatero-Belinchón *et al.*, "Fluvastatin mitigates SARS-CoV-2 infection in human lung cells," *iScience*, vol. 24, no. 12, 2021, doi: 10.1016/j.isci.2021.103469.

[6] Z. R. Zhang *et al.*, "Berbamine hydrochloride potently inhibits SARS-CoV-2 infection by blocking S protein-

mediated membrane fusion," *PLoS Negl. Trop. Dis.*, vol. 16, no. 4, 2022, doi: 10.1371/JOURNAL.PNTD.0010363.

[7] Y. Yan *et al.*, "Bafilomycin A1 induces caspase-independent cell death in hepatocellular carcinoma cells via targeting of autophagy and MAPK pathways," *Sci. Reports 2016 61*, vol. 6, no. 1, pp. 1–13, Nov. 2016, doi: 10.1038/srep37052.

[8] C. A. Fuzo *et al.*, "Celastrol: A lead compound that inhibits SARS-CoV-2 replication, the activity of viral and human cysteine proteases, and virus-induced IL-6 secretion," 2022, doi: 10.1002/ddr.21982.

[9] Y. Chen, M. X. Li, G. D. Lu, H. M. Shen, and J. Zhou, "Hydroxychloroquine/Chloroquine as Therapeutics for COVID-19: Truth under the Mystery," *Int. J. Biol. Sci.*, vol. 17, no. 6, p. 1538, 2021, doi: 10.7150/IJBS.59547.

[10] J. Theobald *et al.*, "In vitro metabolic activation of vitamin D3 by using a multi-compartment microfluidic liver-kidney organ on chip platform," *Sci. Rep.*, vol. 9, no. 1, Dec. 2019, doi: 10.1038/S41598-019-40851-9.

[11] Y. Feng *et al.*, "Exo1: a new chemical inhibitor of the exocytic pathway," *Proc. Natl. Acad. Sci. U. S. A.*, vol. 100, no. 11, pp. 6469–6474, May 2003, doi: 10.1073/PNAS.0631766100.

[12] W. Wu, Y. Wang, X. L. Deng, H. Y. Sun, and G. R. Li, "Cholesterol Down-Regulates BK Channels Stably Expressed in HEK 293 Cells," *PLoS One*, vol. 8, no. 11, p. 79952, Nov. 2013, doi: 10.1371/JOURNAL.PONE.0079952.

[13] D. Chan *et al.*, "Belinostat and panobinostat (HDACI): in vitro and in vivo studies in thyroid cancer," *J Cancer Res Clin Oncol*, vol. 139, pp. 1507–1514, 2013, doi: 10.1007/s00432-013-1465-6.

[14] S. Neelima, P. D. Reddy, C. Sekhar, and K. Bannoth, "Nephroprotective activity of Annona Squamosa leaves against paracetamol-induced nephrotoxicity in rats: in vitro and in vivo experiments," *Futur. J. Pharm. Sci. 2020 61*, vol. 6, no. 1, pp. 1–8, Dec. 2020, doi: 10.1186/S43094-020-00149-4.

[15] L. Li *et al.*, "Histone deacetylase inhibitor sodium butyrate suppresses DNA double strand break repair induced by etoposide more effectively in MCF-7 cells than in HEK293 cells," *BMC Biochem.*, vol. 16, no. 1, pp. 1–9, Jan. 2015, doi: 10.1186/S12858-014-0030-5/FIGURES/5.

[16] N. Sanjib Banerjee, D. W. Moore, T. R. Broker, and L. T. Chow, "Vorinostat, a pan-HDAC inhibitor, abrogates productive HPV-18 DNA amplification," *Proc. Natl. Acad. Sci. U. S. A.*, vol. 115, no. 47, pp. E11138–E11147, Nov. 2018, doi: 10.1073/PNAS.1801156115.
